# Super-Toughened Fumed-Silica-Reinforced Thiol-Epoxy Composites Containing Epoxide-Terminated Polydimethylsiloxanes

**DOI:** 10.3390/ijms22158097

**Published:** 2021-07-28

**Authors:** Goseong Bok, Gayoung Lim, Mingi Kwak, Youngmin Kim

**Affiliations:** Display Research Center, Korea Electronics Technology Institute, 25 Saenariro, Bundang-gu, Seongnam 13509, Korea; bok0215b@naver.com (G.B.); addzero@kakao.com (G.L.); kwakmg@keti.re.kr (M.K.)

**Keywords:** toughness, impact strength, epoxy composite, epoxide-terminated polydimethylsiloxane, fumed silica

## Abstract

In response to the demand for high-performance materials, epoxy thermosetting and its composites are widely used in various industries. However, their poor toughness, resulting from the high crosslinking density of the epoxy network, must be improved to expand their application to the manufacturing of flexible products. In this study, ductile epoxy thermosetting was produced using thiol compounds with functionalities of 2 and 3 as curing agents. The mechanical properties of the epoxy were further enhanced by incorporating fumed silica into it. To increase the filler dispersion, epoxide-terminated polydimethylsiloxane was synthesized and used as a composite component. Thanks to the polysiloxane–silica interaction, the nanosilica was uniformly dispersed in the epoxy composites, and their mechanical properties improved with increasing fumed silica content up to 5 phr (parts per hundred parts of epoxy resin). The toughness and impact strength of the composite containing 5 phr nanosilica were 5.17 (±0.13) MJ/m^3^ and 69.8 (±1.3) KJ/m^2^, respectively.

## 1. Introduction

Owing to their high density in crosslinking [1], epoxy thermosetting resins feature excellent mechanical properties, thermal stability, and chemical resistance. Therefore, they are widely used as the materials for light-weighted composites, as well as high-performance adhesives, sealants, and coating materials in various industries from automobile manufacturing to building construction [2,3,4,5]. Despite their merits, the brittleness of epoxy resins, stemming from the dense crosslinking, impedes their application in flexible products. To overcome this, much effort has been made to endow epoxy resins with ductility by admixing functionalized liquid rubbers [6]. Given that a crack initiates and its tip meets the rubber particles in an epoxy composite, the stress is concentrated on the rubber particles, leading to their cavitation followed by shear deformation; thus, the applied energy is dissipated, and the fracture toughness of the rubber-modified epoxy is enhanced [7]. The toughness of epoxy resins is also enhanced by incorporating high-molecular-weight components into the epoxy formulation, because of the reduction in crosslinking density [8,9]. Therefore, the increase in deformability of the epoxy composites leads to increased toughness. In this study, a ductile epoxy thermosetting was attained by curing bisphenol A diglycidylether (BPDGE) with a thiol curing agent [10,11,12]. The thiol curing agent consisted of thiol-based compounds with functionalities of 2 and 3 at the weight ratio of 1 to 1. Because of the character of the thiol structure [10], the epoxy thermosetting produced by the epoxy-thiol click reaction is ductile (Figure S1 and Table 1). In addition, the mechanical properties of the thermosetting are further optimized by incorporating fillers into it. Given that the mechanical properties of composites are enhanced by the strong interfacial interactions between fillers and the matrix [13], the filler-loading technique is crucial to the realization of high-performance composites. Among fillers, nanosilica is widely used to fabricate nanocomposites because of its high reinforcing effectiveness [14,15] and low price. However, the agglomeration of the nanosilica induced by the hydrogen bonding between the silanol groups of silica deteriorates the mechanical properties and processability of the resulting composites. To overcome this, a novel epoxide-terminated polydimethylsiloxane (ep-PDMS) was synthesized and admixed with BPDGE to increase the dispersion efficiency of fumed silica in this study. The interaction between the polysiloxane and silanol groups of silica was previously studied through calculation [16] and experiments [13,17,18]. After confirming by scanning electron microscopy that ep-PDMS enhanced the dispersion of fumed silica in epoxy-based composite, the effect of the fumed silica content on the rheological properties, mechanical properties, impact strength, and thermal stability of the composites was investigated.

## 2. Results and Discussion

**Figure 1:**
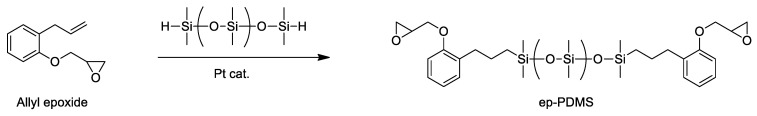
Synthesis of ep-PDMS.

Epoxide-terminated polydimethylsiloxane (ep-PDMS) was synthesized by reacting an allyl epoxide with a hydride-terminated PDMS through the platinum-catalyzed hydrosilylation reaction [19] (Figure 1). The completion of the reaction was ascertained by the disappearance of the IR absorption at 2125 cm^−1^ for Si–H stretching [20] (Figure S2). NMR spectroscopy was conducted to characterize ep-PDMS. In the ^1^H NMR spectrum of ep-PDMS, the proton resonances for Si–CH_2_ formed via the hydrosilylation reaction appeared at around 0.6 ppm. The proton peaks for oxirane were observed at 2.9 ppm and 3.6 ppm, indicating that oxirane was intact despite the hydrosilylation reaction. The presence of siloxanes was confirmed by the two signals observed at 7.59 ppm (for CH_2_Si(CH_3_)_2_O) and −21.80 ppm (for OSi(CH_3_)_2_O) in the ^29^Si NMR spectrum of ep-PDMS.

**Figure 2:**
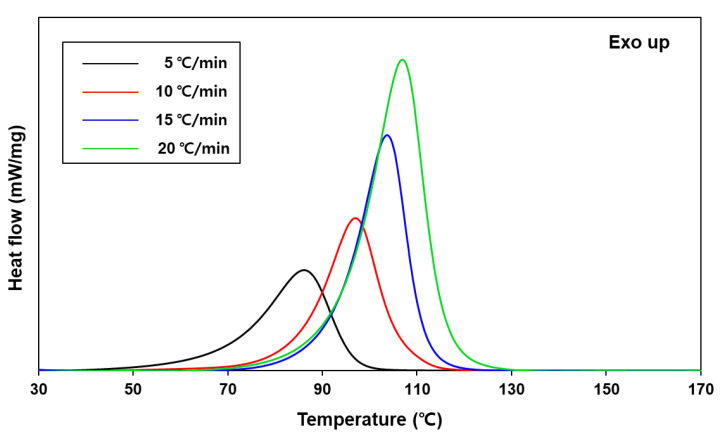
DSC thermograms of epoxy/thiol/MI system obtained at various heating rates.

Having produced this compound, the epoxy composition was fabricated by admixing ep-PDMS and BPDGE at the ep-PDMS-to-BPDGE weight ratio of 1 to 9. Then, a stoichiometric amount of thiols and a catalytic amount of 1-methylimidazole (MI) were added to the epoxy resin. The thiol-based curing agent comprised EDT and TMPMP with functionalities of 2 and 3, respectively, at the EDT-to-TMPMP weight ratio of 1 to 1. Because the base-catalyzed epoxy-thiol curing reaction is complex [11], the degree of curing obtained from the kinetic parameters (apparent activation energy, pre-exponential factor, and reaction order) was used to determine the process conditions. The curing behavior of the epoxy/thiol/MI system was investigated by non-isothermal differential scanning calorimetry (DSC) at various heating rates (*β*). When the samples were heated in a differential scanning calorimeter, one exothermic peak stemming from epoxy curing was observed, and the peak temperature (*T_p_*) of the exothermic curve increased with increasing *β* (Figure 2). 

**Figure 3:**
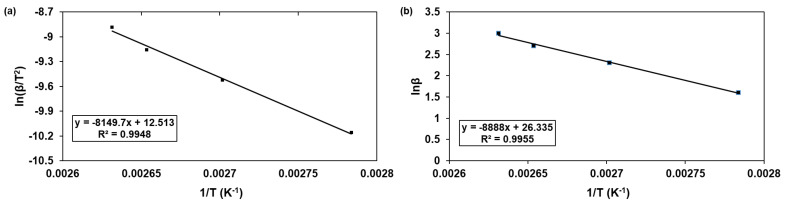
(**a**) Kissinger plot and (**b**) Ozawa plot of epoxy/thiol/MI system.

After data obtained from the DSC analysis were converted to ln(*β*/*T_p_*^2^) and 1/*T_p_*, the apparent activation energy (*E_a_*) and pre-exponential factor (A) were calculated based on the Kissinger equation (Equation (1)) [21] (Figure 3a). The *E_a_* and A values of the epoxy/thiol/MI curing were calculated to be 67.76 KJ/mol and 2.26 × 10^8^ min^−1^ from the slope and y-intercept of the plotting line of 1/*T_p_* vs. ln(*β*/*T_p_*^2^). The apparent activation energy was also calculated based on the Ozawa equation (Equation (2)) [22] (Figure 3b). The linear relation between 1/*T_p_* and ln(*β*) was obtained, and the *E_a_* value was calculated to be 70.25 KJ/mol from the slope of the line.

(1) $$\ln\left(\frac{\beta }{T_{p}^{2}}\right)=\ln\left(\frac{AR}{E_{a}}\right)-\frac{E_{a}}{RT_{p}}$$

(2) $$\ln\left(\beta \right)=\ln\left(\frac{AE_{a}}{R}\right)-\ln\;F\left(a\right)-5.331-1.052\frac{E_{a}}{RT}$$

(3) $$\frac{d\left(ln\beta \right)}{d\left(\frac{1}{T_{p}}\right)}=\frac{-E_{a}}{nR}$$

(4) $$\alpha =1-{\left[1+\left(n-1\right)\cdot A\cdot \exp\left(-\frac{E_{a}}{RT}\right)\cdot t\right]}^{\frac{1}{1-n}}$$

where *β* is the heating rate, K/min; *T_p_* is the exothermic peak temperature, K; *E_a_* is the apparent activation energy, J/mol; *R* is the perfect gas constant of 8.314 J/(mol·K); and *F*(*a*) is a constant function.

**Figure 4:**
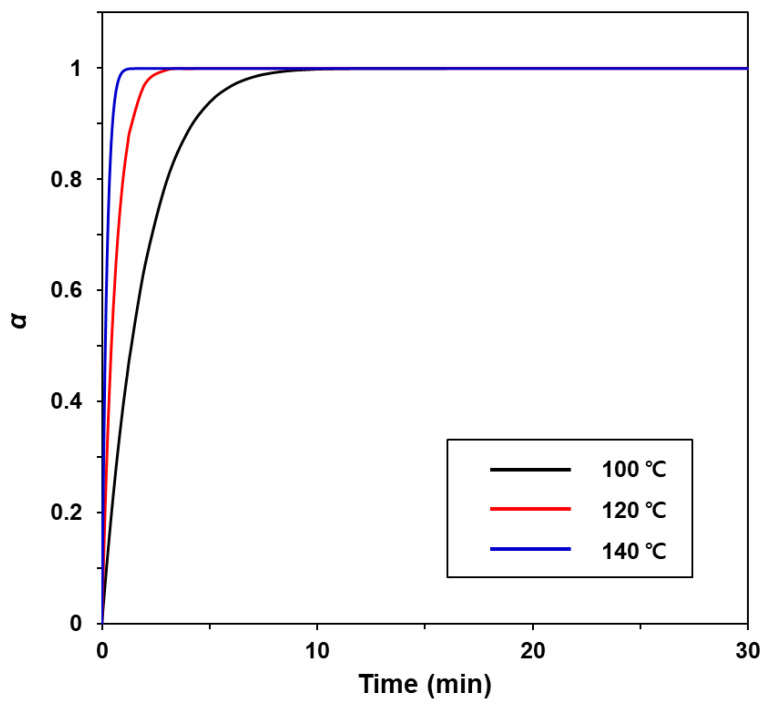
Degree of curing of epoxy/thiol/MI system over time at various temperatures.

Next, the reaction order (*n*) of the epoxy/thiol/MI curing was determined as 0.93 using the averaged *E_a_* value of 69.00 KJ/mol according to the simplified Crane equation (Equation (3)) [21]. Finally, the degree of curing (*α*) of the epoxy/thiol/MI system over time at various temperatures was obtained based on Equation (4) [21], and the results are shown in Figure 4. The curing degree of the epoxy/thiol/MI system increased with increasing temperature and extended curing time. For the next step, the epoxy/thiol/MI system was designated as neat epoxy.

**Figure 5:**
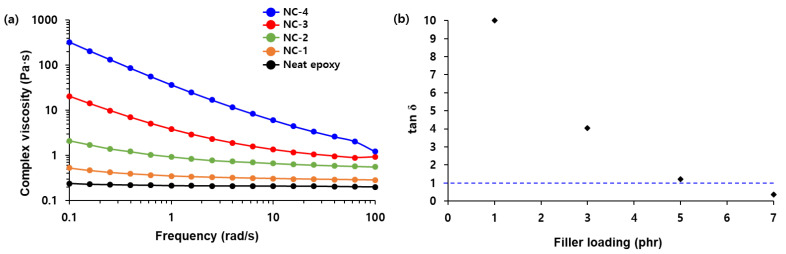
(**a**) Shear-rate dependency of the complex viscosity of neat epoxy and the fumed silica composites and (**b**) the filler loading dependence of tan δ measured at 1 rad/s.

Next, nanocomposites were fabricated by mixing neat epoxy and fumed silica. Thanks to the possible interactions between the (Si–O) backbone of ep-PDMS and the silanol groups of the nanosilica, the filler loading reached 7 phr (parts per hundred parts of epoxy) in the composites. The effect of nanosilica loading on the rheological properties of the composites was investigated by measuring the frequency dependence of complex viscosity (Figure 5a). The complex viscosity of the composites increased with increasing nanosilica content. While the complex viscosity of neat epoxy and NC-1 was independent of the frequency (Newtonian flow), that of NC-2, NC-3, and NC-4 decreased with increasing shear rate (shear thinning). This can be explained by the fact that particles align with the flow direction to weaken the particle–particle and polymer–particle interactions at a high shear rate. Therefore, the flow resistance caused by the clusters of fumed silica decreased, leading to low viscosity at a high shear rate [23,24], and this shear-thinning character was more noticeable in the composites with high filler loading. Figure 5b shows the loss factor (tan δ) of neat epoxy and the composites measured at 1 rad/s. While tan δ of NC-4 was less than 1, those of others were higher than 1, indicating that NC-4 behaved like a solid due to the high filler loading. Given that high viscosity deteriorates the processability of composites, the technique to maximize filler loading while maintaining flowability of composites is crucial.

**Figure 6:**
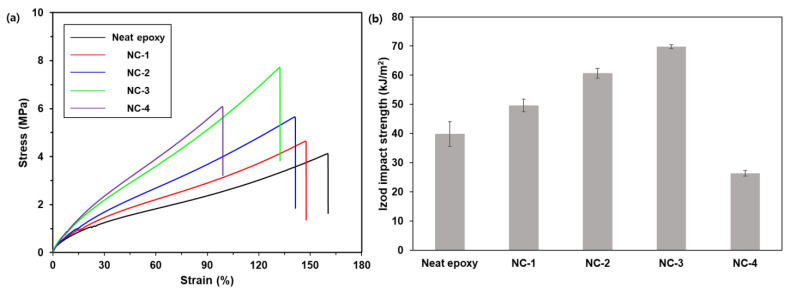
(**a**) Stress-strain curves of neat epoxy and nanocomposites and (**b**) Izod impact strength of nanocomposites.

The effect of the nanosilica content on the mechanical properties of the nanocomposites was investigated through a tensile test. Dog-bone-shaped specimens were pulled in the tensile direction until they were broken. The mechanical properties are summarized in Table 1, and stress-strain curves are shown in Figure 6a. While the tensile strength, Young’s modulus, and toughness of the composites increased with increasing fumed silica content up to 5 phr, elongation at break decreased, though the change in the latter was smaller. Compared to neat epoxy, the strength, modulus, and toughness of NC-3 increased by 76%, 50%, and 44%, respectively, and the elongation decreased by 15%. The toughness of NC-3 reached 5.17 (±0.13) MJ/m^3^. It is interesting that the strength and toughness of NC-4 were lower than those of NC-3 despite higher filler loading. Given that clusters of fumed silica aggregates were formed with increased filler content, the density in the filler-rich phase was high in NC-4; thus, the applied energy during the tensile test was concentrated on the fillers and not transferred to the matrix, causing fracture to occur at the low elongation.

Next, to investigate the effect of ep-PDMS on the mechanical properties of the composites, a control composite containing 5 phr fumed silica was fabricated without ep-PDMS. The mechanical properties of the control sample are summarized in Table S1, and its stress-strain is shown in Figure S3. Compared to the control composite, the strength, elongation, and toughness of NC-3 were higher by 28%, 43%, and 53%, respectively, with the same filler content, suggesting that ep-PDMS is effective to disperse fumed silica in epoxy resins. The poor dispersion of fumed silica in the control sample was confirmed by the FESEM images (Figure S4). Unlike NC-3, micron-sized clusters of fumed silica were clearly observed in the control composite.

**Figure 7:**
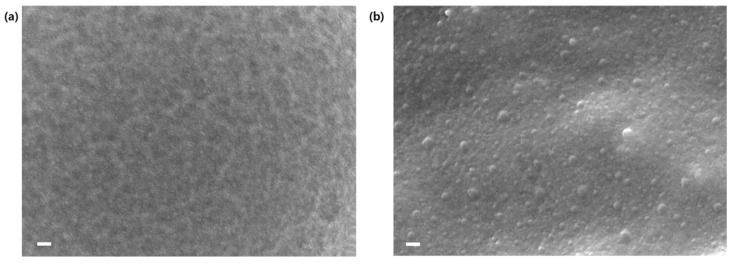
FESEM images of the fractured surface of (**a**) NC-3 and (**b**) NC-4 after a notched Izod impact test. A scale bar represents 100 nm.

The effect of fumed silica content on the impact strength of composites was investigated through a notched Izod impact test. Rectangular specimens with a V-shaped notch were struck by a hammer, and the absorbed energy was recorded. The results are summarized in Table 1 and shown in Figure 6b. The impact strength of the composites increased with an increasing nanosilica content ranging from 0 to 5 phr. It is interesting that the specimens were partially broken after the Izod impact test, indicating that they were ductile. Compared to neat epoxy, the impact strength of NC-3 increased by 75%, and this enhancement was attributed to the strong filler–matrix interaction with the help of ep-PDMS. It should be noted that the impact strength of NC-3 was about 70 KJ/m^2^, and this high value is rare for epoxy composites [25,26,27,28,29,30] (Figure S5) and comparable to those of super-toughened poly(lactic acid) blends with impact strength higher than 53 KJ/m^2^ [31]. On the contrary, the impact strength of NC-4 was even lower than that of neat epoxy by 34%, implying that the presence of filler-rich phase led to severe deterioration in the impact strength of the composites despite high filler loading. Given that NC-4 was completely broken after the Izod impact test, whereas other samples were partially broken, the clusters of fumed silica endowed NC-4 with brittleness. The surface morphologies of fractured NC-3 and NC-4 after the Izod impact test were analyzed by FESEM (Figure 7). While the surface of NC-3 was smooth, filler-rich phase with bulging nanosilica was observed on that of NC-4. 

**Figure 8:**
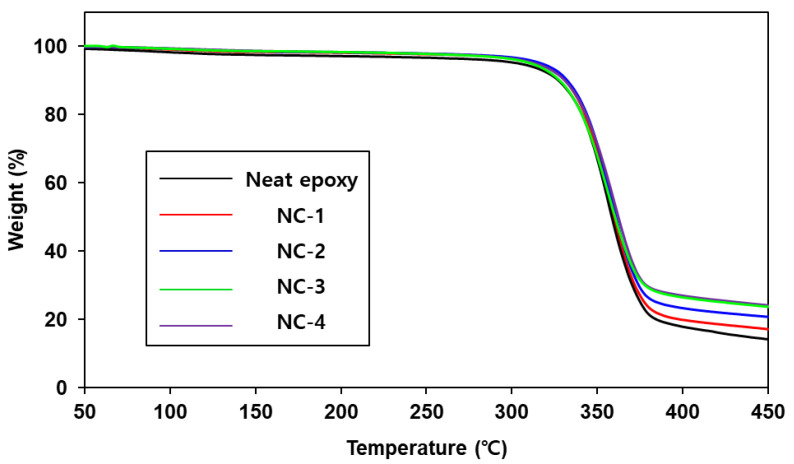
TGA thermograms of neat epoxy and nanocomposites.

Next, the thermal stability of the neat epoxy and the nanocomposites was investigated by thermogravimetric analysis (TGA) in temperatures ranging from 50 °C to 450 °C (Figure 8). All specimens were stable up to 300 °C, at which the weight loss percentage was less than 5%. In addition, the amount of the residue at 450 °C increased with increasing filler loading in the composites. DSC measurements of them were carried out to determine the glass transition temperature (Tg), and the results are summarized in Table 2. The Tg values of the composites also increased with increasing fumed silica loading, though the change was little.

## 3. Materials and Methods

### 3.1. Materials

Bisphenol A diglycidyl ether (BPDGE, EEW = 190 g/eq) was purchased from Kukdo Chemical Co., Ltd (Seoul, Korea). 1-methylimidazole (MI) was purchased from Tokyo Chemical Industry Co., Ltd. (Tokyo, Japan). Hydride-terminated poly(dimethylsiloxane) (Mn~580, h-PDMS), platinum(0)-1,3-divinyl-1,1,3,3-tetramethyldisiloxane complex solution in xylene (Pt cat), 2,2′-(ethylenedioxy)diethanethiol (EDT), trimethylolpropane tirs(3-mercaptopropioante) (TMPMP), and anhydrous magnesium sulfate were purchased from Sigma-Aldrich Korea Ltd. (Yongin, Korea). Toluene was purchased from Samchun Chemical (Seoul, Korea). Fumed silica (K-200) of silica aggregates consisting of 7 to 40 nm primary particles was purchased from OCI (Seoul, Korea). All chemicals were used as received without purification.

### 3.2. Instrumentation

The ^1^H NMR, ^13^C NMR, and ^29^Si NMR spectra were measured on an NMR spectrometer equipped with Bruker Top Spin 3.2 software (Ascend™ 400, Bruker, Madison, WI, USA). The Fourier transform infrared (FTIR) spectra in the range from 500 to 4000 cm^−1^ were obtained through the attenuated total reflectance method using an FTIR spectrophotometer (IRAffinity-1S, Shimadzu, Kyoto, Japan). A tensile test was carried out using a universal testing machine (HZ-1003A/B(1T), MMS Tech, Bucheon, Korea). A non-isothermal DSC analysis was performed using a DSC-4000 (PerkinElmer, Waltham, MA, USA). Izod impact strength was measured using an Izod impact tester (KP-M3940D, KIPAE). The surface morphologies of the fractured composites were analyzed using a field emission scanning electron microscope (FESEM). 

### 3.3. Synthesis of ep-PDMS 

An allyl epoxide was synthesized following the previously reported method [32]. A flask was charged with allyl epoxide (6.0 g, 31.58 mmol), h-PDMS (9.16 g, 15.78 mmol), Pt cat (0.16 g), and toluene (15.16 g). This solution was heated at 95 °C using an oil bath. After 12 h stirring, the mixture was poured into a separatory funnel and sequentially washed with deionized water and brine solution. The organic layer was separated and dried over anhydrous magnesium sulfate. The organic layer was filtered through celite, and all volatiles were removed from the filtrate under reduced pressure to produce ep-PDMS in a brown liquid with the viscosity of 72 mPa·s at 25 °C. The epoxy equivalent of ep-PDMS was calculated as 480 g/eq. 

^1^H NMR (400 MHz, CDCl_3_) data: δ: 7.15–7.13 (m, aromatic ring), 6.93–6.90 (t, aromatic ring), 6.83–6.81 (d, aromatic ring), 4.25–3.80 (d, O-CH_2_), 3.66–3.62 (m, glycidyl), 2.90–2.88 (d, glycidyl), 2.78–2.65 (t, ArCH_2_), 1.66 (m, SiCH_2_CH_2_), 0.75–0.55 (t, SiCH_2_), 0.30–0.00 (m, SiCH_3_).

^13^C NMR (100 MHz, CDCl_3_) data: δ: 156.4, 131.5, 130.1, 126.9, 121.0, 111.4, 68.6, 50.4, 44.6, 34.0, 23.7, 18.4, 0.99, 0.79.

^29^Si NMR (79 MHz, CDCl_3_) data: δ: 7.59 (CH_2_Si(CH_3_)_2_O), −21.80 (OSi(CH_3_)_2_O).

### 3.4. Preparation of Compositions of Neat Epoxy and Nanocomposites

Each composition of neat epoxy and nanocomposites is summarized in Table 3. A 50 mL vial was charged with BPDGE, ep-PDMS, and fumed silica, and the mixture was subject to an ultra-sonication treatment. After 2 h, EDT, TMPMP, and MI were added to the mixture. Each composition was agitated using a vortex mixer for 20 min and degassed under reduced pressure.

### 3.5. Tensile Test

The as-prepared compositions were poured into a dog-bone-shaped Teflon mold and heated at 140 °C for 1 h. Then, the specimens were placed in a tensile tester and pulled along the z-axis until they were broken.

### 3.6. Izod Impact Strength Test

Izod impact strength was measured following an ASTM D256 standard test method using a pendulum-C type hammer. The as-prepared compositions of neat epoxy and nanocomposites were poured into a Teflon mold with dimensions of 63.5 mm × 12.7 mm × 6.35 mm (width × length × thickness) and kept at 140 °C in a convection oven. After 1 hour, all specimens were cooled to room temperature, and a V-notch with a depth of 2.54 mm was made at the center of them using a cutter.

## 4. Conclusions

In this work, epoxide-terminated PDMS (ep-PDMS) was synthesized and used to produce thiol-epoxy composites containing fumed silica. Thanks to the polysiloxane–silica interaction, the mechanical properties of the epoxy composites containing ep-PDMS improved with increasing fumed silica content up to 5 phr. The toughness and impact strength of NC-3 were 5.17 (±0.13) MJ/m^3^ and 69.8 (±1.3) KJ/m^2^, respectively. The mechanical properties of the composite without ep-PDMS containing 5 phr nanosilica were inferior to those of NC-3 despite having the same filler content because of the poor dispersion of fumed silica. In conclusion, the employment of ep-PDMS is expected to be an effective way to realize super-toughened fumed-silica epoxy composites.

## Figures and Tables

**Table 1:** Mechanical properties of neat epoxy and nanocomposites.

	Neat Epoxy	NC-1	NC-2	NC-3	NC-4
Tensile strength (MPa)	4.2 ± 0.3	4.8 ± 0.6	5.7 ± 0.6	7.4 ± 0.3	5.7 ± 0.5
Elongation at break (%)	156 ± 8	147 ± 20	142 ± 2	133 ± 3	99 ± 11
Young’s modulus (MPa)	10.3 ± 0.5	11.3 ± 0.3	13.9 ± 0.8	15.4 ± 1.1	16.7 ± 0.2
Toughness (MJ/m^3^)	3.6 ± 0.02	3.8 ± 0.88	4.35 ± 0.42	5.17 ± 0.13	3.14 ± 0.15
Impact strength (KJ/m^2^)	39.9 ± 8.5	49.7 ± 4.3	60.7 ± 3.4	69.8 ± 1.3	26.4 ± 1.8

**Table 2:** Glass transition temperature of neat epoxy and nanocomposites.

	Neat Epoxy	NC-1	NC-2	NC-3	NC-4
Tg (°C)	11.24	12.30	12.31	12.45	12.75

**Table 3:** The compositions of neat epoxy and nanocomposites.

	Neat Epoxy	NC-1	NC-2	NC-3	NC-4
BPDGE (g)	9	9	9	9	9
ep-PDMS (g)	1	1	1	1	1
EDT (g)	2.67	2.67	2.67	2.67	2.67
TMPMP (g)	2.67	2.67	2.67	2.67	2.67
MI (g)	0.11	0.11	0.11	0.11	0.11
Fumed silica (g)	0	0.1	0.3	0.5	0.7
Filler loading (phr) ^a^	0	1	3	5	7

^a^ phr—parts per hundred parts of epoxy resin.

## Data Availability

Data sharing not applicable.

## Supplementary Materials

The following are available online at https://www.mdpi.com/1422-0067/22/15/8097/s1.
