# Correction: Bok et al. Super-Toughened Fumed-Silica-Reinforced Thiol-Epoxy Composites Containing Epoxide-Terminated Polydimethylsiloxanes. *Int. J. Mol. Sci.* 2021, *22*, 8097

**DOI:** 10.3390/ijms23158545

**Published:** 2022-08-01

**Authors:** Goseong Bok, Gayoung Lim, Mingi Kwak, Youngmin Kim

**Affiliations:** Display Research Center, Korea Electronics Technology Institute, 25 Saenariro, Bundang-gu, Seongnam 13509, Korea; bok0215b@naver.com (G.B.); addzero@kakao.com (G.L.); kwakmg@keti.re.kr (M.K.)

The authors wish to make the following corrections to the original publication [1]. In the original publication, there were mistakes in Table 1, mechanical properties of neat epoxy and nanocomposites, and Table S1, mechanical properties of the BPDGE/thiol/MI system and control composite, published. The reason for this correction is that the toughness values in Tables 1 and S1 were inadvertently calculated by using a percentage of the strain shown in Figures 6a, S1, and S3, which resulted in erroneous values. The corrected Table 1 and Table S1, calculated using the strain (dimensionless), appear below. 

There were errors in the original publication:

(1) In the Abstract

“The toughness and impact strength of the composite containing 5 phr nanosilica were 517 (±13) MJ/m^3^ and 69.8 (±1.3) KJ/m^2^, respectively” 

should be changed to

“The toughness and impact strength of the composite containing 5 phr nanosilica were 5.17 (±0.13) MJ/m^3^ and 69.8 (±1.3) KJ/m^2^, respectively”.

(2) In page 4–5

“The toughness of NC-3 reached 517 (±13) MJ/m^3^” should be changed to “The toughness of NC-3 reached 5.17 (±0.13) MJ/m^3^”.

(3) In Conclusions 

“The toughness and impact strength of NC-3 were 517 (±13) MJ/m^3^ and 69.8 (±1.3) KJ/m^2^, respectively“ should be changed to “The toughness and impact strength of NC-3 were 5.17 (±0.13) MJ/m^3^ and 69.8 (±1.3) KJ/m^2^, respectively”.

The authors apologize for any inconvenience caused and state that this correction does not change the impact strength (69.8 ± 1.3 KJ/m^2^) of NC-3. Given that this is higher than the impact strength of 53 KJ/m^2^ (a criterion for super toughness in page six), the correction has no material impact on the scientific results of our paper. This correction was approved by the academic editor. The original publication has also been updated.

## Figures and Tables

**Table 1:** Mechanical properties of neat epoxy and nanocomposites.

	Neat Epoxy	NC-1	NC-2	NC-3	NC-4
Tensile strength (MPa)	4.2 ± 0.3	4.8 ± 0.6	5.7 ± 0.6	7.4 ± 0.3	5.7 ± 0.5
Elongation at break (%)	156 ± 8	147 ± 20	142 ± 2	133 ± 3	99 ± 11
Young’s modulus (MPa)	10.3 ± 0.5	11.3 ± 0.3	13.9 ± 0.8	15.4 ± 1.1	16.7 ± 0.2
Toughness (MJ/m^3^)	3.6 ± 0.02	3.8 ± 0.88	4.35 ± 0.42	5.17 ± 0.13	3.14 ± 0.15
Impact strength (KJ/m^2^)	39.9 ± 8.5	49.7 ± 4.3	60.7 ± 3.4	69.8 ± 1.3	26.4 ± 1.8

**Table S1:** Mechanical properties of the BPDGE/thiol/MI system and control composite.

	BPDGE/Thiol/MI	Control Composite
Tensile strength (MPa)	5.2 ± 0.4	5.8 ± 0.3
Elongation at break (%)	143 ± 3.8	93 ± 5.9
Young’s modulus (MPa)	12.8 ± 0.4	25.9 ± 0.3
Toughness (MJ/m^3^)	4.48 ± 0.16	3.37 ± 0.55
Impact strength (KJ/m^2^)	40.2 ± 2.0	26.0 ± 1.2
