# Supplementary material for: Super-Toughened Fumed-Silica-Reinforced Thiol-Epoxy Composites Containing Epoxide-Terminated Polydimethylsiloxanes
Source: Int J Mol Sci. 2021 Jul 28;22(15):8097. doi: 10.3390/ijms22158097 (PMC8347240; doi:10.3390/ijms22158097)
Supplement: Supplementary file 1 [file ijms-22-08097-s001.zip › ijms-1313752-supplementary.pdf]

International Journal of
Molecular Sciences

MDPI

Supplementary Materials

Super-Toughened Fumed-Silica-Reinforced Thiol-Epoxy Composites Containing Epoxide-Terminated Polydimethylsiloxanes

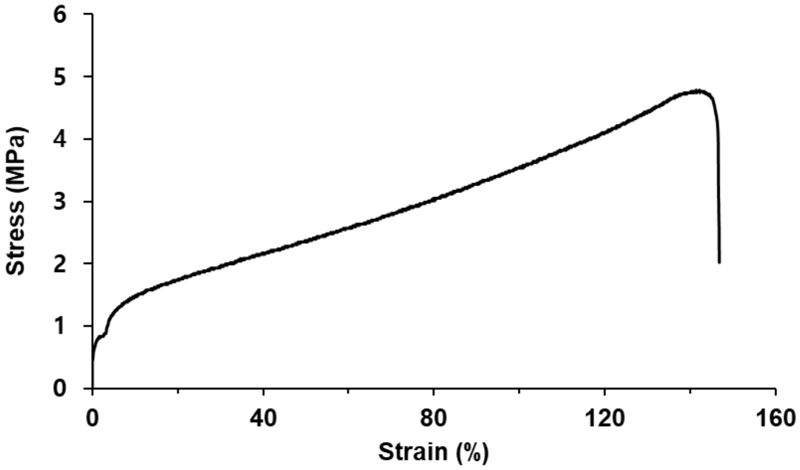

Figure S1. A stress-strain curve of BPDGE/thiol/MI.

Table S1. Mechanical properties of the BPDGE/thiol/MI system and control composite.

	**BPDGE/thiol/MI**	**Control composite**
Tensile strength (MPa)	5.2 ± 0.4	5.8 ± 0.3
Elongation at break (%)	143 ± 3.8	93 ± 5.9
Young's modulus (MPa)	12.8 ± 0.4	25.9 ± 0.3
Toughness (MJ/m^3)	4.48 ± 0.16	3.37 ± 0.55
Impact strength (KJ/m^2)	40.2 ± 2.0	26.0 ± 1.2

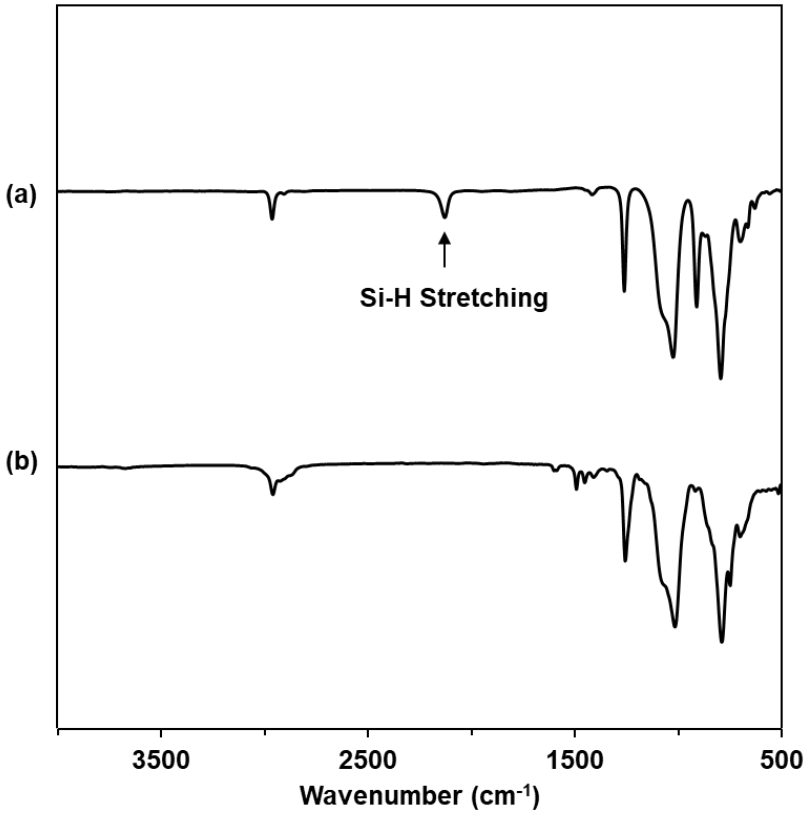

Figure S2. FTIR spectra of (a) h-PDMS and (b) ep-PDMS.

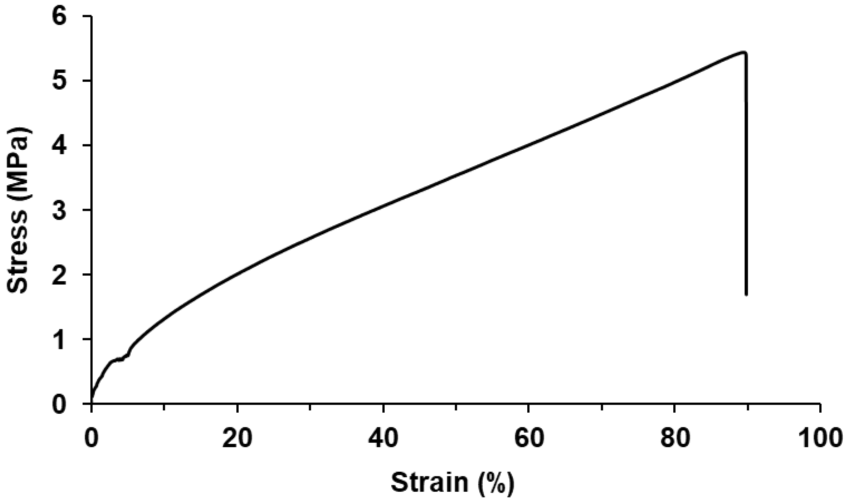

Figure S3. A stress-strain curve of the control composite.

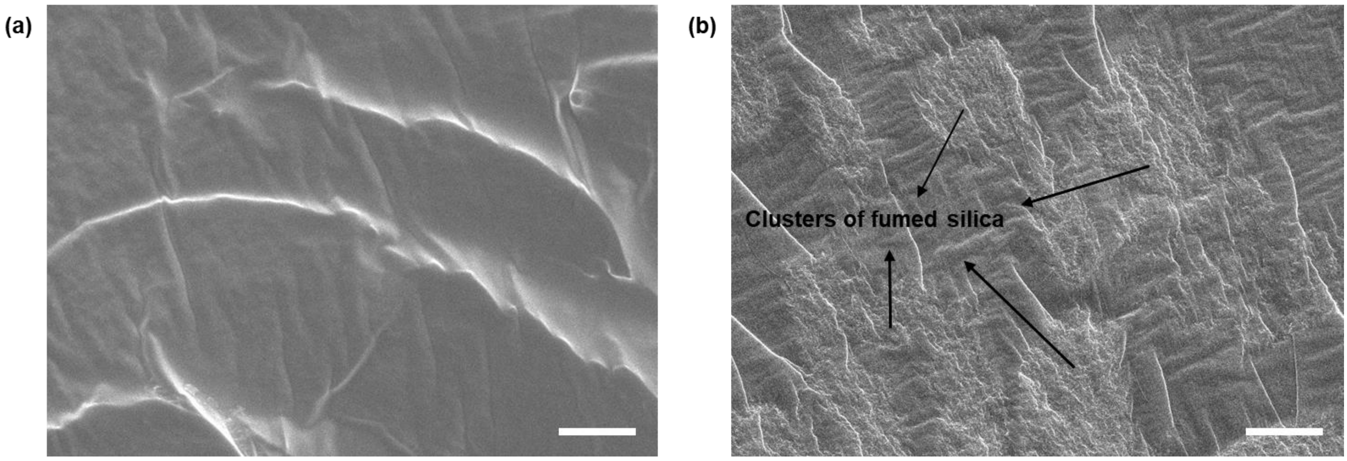

Figure S4. FESEM images of the fractured surfaces of (a) NC-3 and (b) the control composite. A scale bar represents 10 μm.

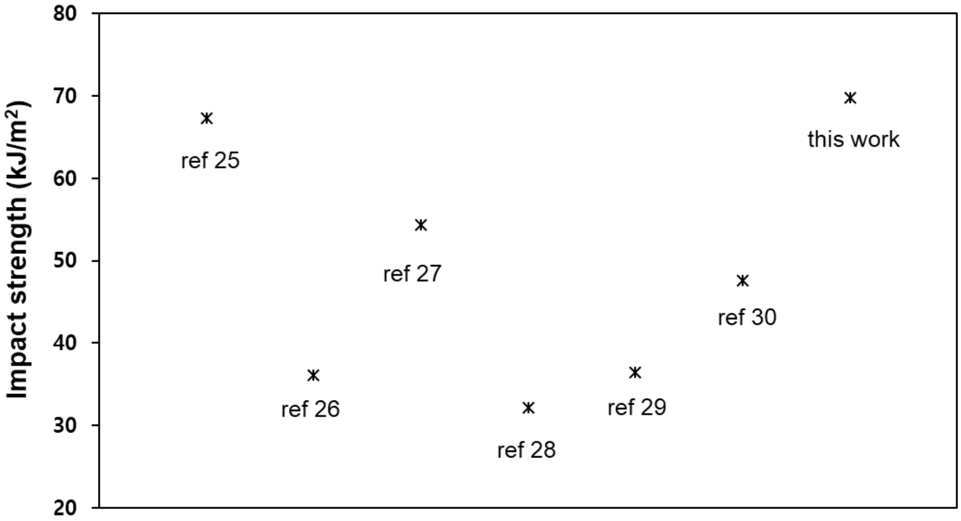

Figure S5. Selected reinforced epoxy composites and their maximum impact strength.
